# Engineering Solutions to COVID-19 and Racial and Ethnic Health Disparities

**DOI:** 10.1007/s40615-020-00953-x

**Published:** 2021-02-10

**Authors:** Gilda A. Barabino

**Affiliations:** Biomedical and Chemical Engineering, Olin College of Engineering, Olin Way, Needham, MA 02492 USA

**Keywords:** Engineering, Health disparities, COVID-19, User-centered solutions, Systems problems

## Abstract

The role of engineers in response to the COVID-19 pandemic and in the elimination of health disparities, while not always visible, has important implications for the attainment of impactful solutions. The design skills, systems approach, and innovative mindset that engineers bring all have the potential to combat crises in novel and impactful ways. When a disparities lens is applied, a lens that views gaps in access, resources, and care, the engineering solutions are bound to be more robust and equitable. The disproportionate impact of COVID-19 on the Black community and other communities of color is linked to inequities in health rooted in a centuries long structural racism. Engineers working collaboratively with physicians and healthcare providers are poised to close equity gaps and strengthen the collective response to COVID-19 and future pandemics.

## Introduction

Engineers as capable and necessary responders to pandemics has been demonstrated throughout the ages. The design skills, systems approach, and innovative mindset that engineers bring all have the potential to combat crises in novel and impactful ways. And when a disparities lens is applied, a lens that views gaps in access, resources, and care, the engineering solutions are bound to be more robust and equitable. Equipping engineers to effectively apply a disparities lens requires them to understand the complexities and implications of racial disparities to include the public health concerns and the historical context of differential medical treatment based on race. The disproportionate impact of COVID-19 on Black communities and other communities of color has increased the need for engineers equipped to develop user-centered solutions that can address racial disparities.

According to the Johns Hopkins Coronavirus Resource Center, as of October 11, 2020, the number of cases of COVID-19 in the United States is over 7.8 million and the number of deaths is over 214,000 [1]. When analyzing the COVID-19 reported infections and deaths, the APM Research Lab reports that as of September 15, 2020, the age-adjusted COVID-19 mortality rate for Black Americans is 3.4 times as high as that for whites [2]. This striking statistic is to be expected given the legacy of slavery and its promulgation of inequities in healthcare for Blacks [3]. Blacks suffered disproportionately in a previous influenza pandemic in 1918, and now in the current pandemic, inequities remain as Blacks cannot escape the centuries old ingrained systemic racism in this country. Among the myriad factors contributing to the increased disease burden for Blacks are underlying comorbidities, working and living conditions that impede social distancing, and lack of access to testing and adequate healthcare.

While Blacks make up 13% of the US population, they account for just 5% of the science and engineering workforce, 5% of higher education faculty across all disciplines, 4% of PhDs in engineering, 4% of physicians, and less than 7% of medical students [4–6]. Unfortunately, biases and structural racism contribute to the dearth of Black engineers and physicians as well as poor clinical outcomes for Black Americans linked to health disparities that are further exacerbated by the COVID crisis. Improved outcomes can be obtained by having more Black physicians and healthcare providers, and more Black faculty and engineers, who are more likely to serve their community, understand their needs, and develop effective approaches for treatment and care (https://www.nytimes.com/2020/01/13/upshot/race-and-medicine-theharm-that-comes-from-mistrust.html). Driven by communal values such as altruism, students from underrepresented groups gravitate toward careers in medicine and fields that provide opportunities to have societal impact, such as engineering [7]. Training on health disparities for engineers and others can lead to effective user-centered solutions.

Examining engineering solutions to COVID along with engineering solutions to health disparities affords an opportunity to operate at the intersection to realize better solutions. Many of the ways in which engineers have provided and continue to provide solutions to COVID-19 are described in the following section. In many cases, these solutions would be enhanced with the application of a health disparities lens to ensure that solutions are designed such that all populations are able to receive benefit. Equipping engineers to apply a health disparities lens requires education and practice. Examples are included in the section on engineering solutions to health disparities.

## Engineering Solutions to COVID

Posing the response to COVID-19 as a systems problem, engineers have effectively collaborated with scientists and clinicians to understand, limit spread, and contribute to the development of a novel vaccine and effective therapies. Engineers helped speed up the process of determining the genetic structure of the SAR-Cov-2 virus (severe acute respiratory syndrome coronavirus 2) responsible for COVID-19. They also turned their attention to models and simulations to better understand transmission through aerosol droplet and patterns of transmission. While scientists and clinicians are conducting studies and trials to develop a safe vaccine, in parallel engineers are designing processes to scale up the production of billions of doses. They are also working on the design of manufacturing processes to produce the needed therapeutics and to shore up supply chains needed to get the right materials to the right places where they are needed at the right time. In the area of personal protective equipment (PPE), engineers rose to the challenge in meeting demands, addressing shortages, and finding new devices and materials useful for safety and protection. For example, 3-D printed masks, swabs, and face shields along with 3-D printed parts for devices that were being modified to serve as ventilators helped with unmet needs, particularly during the early stages of the pandemic. For much needed enhancements for testing and surveillance, engineers are behind the rapid development of point-of-care diagnostic tests, employing tools of artificial intelligence (AI), automation, and process control for the production of tests. They are also employing these tools for the development of predictive models simulating real-world conditions.

The National Academy of Engineering, the National Institutes of Health, and the National Science Foundation spearheaded specific engineering initiatives in response to COVID-19 that have been met with enthusiasm and have great potential for the future.

The National Academy of Engineering launched the COVID-19 Call for Engineering Action (https://www.nae.edu/230195/COVID19-Call-for-Engineering-Action), an intergenerational effort connecting its Grand Challenges Scholar Program (aimed at students) and Frontiers of Engineering (aimed at mid-career faculty) communities to collectively brainstorm on innovative engineering approaches to address the COVID-19 crisis and its consequences. Examples of ideas that have emerged in response to this call are paper-based tests for at-home diagnostics, self-disinfecting N-95 masks using copper coatings that can destroy viral pathogens, wearable sensors for monitoring, and handleless door openers using foot-operated devices.

The National Institutes of Health launched RADx “to support the development, production scale-up, and deployment of accurate, rapid tests across the country” [8]. Notably, longer-term, RADx seeks to include, in its rapid production of innovative tests, strategies to ensure that they reach vulnerable and underserved populations. A component of the RADx program, RADx-UP, is aimed at elucidating factors that contribute to the disproportionate COVID-19 disease burden borne by underserved populations and improving access to and effectiveness of testing. RADx anticipates challenges ahead and will be calling on engineers to address them. These challenges include rapid validation processes for promising prototype devices, development of digital platforms for health records and data, and the scale-up of test production along with wide-scale distribution of tests at appropriate locales.

In response to COVID-19, the National Science Foundation was quick to mobilize its RAPID Response Research program to support a host of projects led by engineers and scientists. Beyond its current COVID-19 targeted projects, NSF has a history of previous investments that are being employed in the COVID-19 response such as advanced manufacturing initiatives, fundamental studies that enabled rapid sequencing and identification of the coronavirus, and NSF-supported molecular institutes and super computers.

What these approaches share in common is the mobilization of tools and technologies for speed and scale to address the pandemic. Having engineers equipped through training and practice enhances their ability to respond equitably, and that training must include an understanding of health disparities and engineering approaches to address them.

## Engineering Solutions to Health Disparities

A systems approach to health disparities by engineers affords a special opportunity to merge the development of innovative technologies with unmet health needs fueled by structural racism and social determinants of health. Without a lens through which inequities that shape disease processes and access to care, designs that are accessible and fully effective for all populations will be lacking. Collaborations between engineers and clinicians through clinically centered experiences—also known as clinical immersion and biodesign—allows for the collaborative identification of unmet needs and pursuit of technical solutions to meet those needs. This can be done in a number of training settings ranging from courses co-led by engineering and medical schools to intensive short-term programs. The author has led a program called Coulter College funded by the Wallace H. Coulter Foundation and recently hosted by Medtronic. During an intensive 3-day program focused on the translation of biomedical innovations, student teams are guided through a dynamic process to develop solutions to clinical needs while gaining a better understanding of resource constraints and disparities that must be considered during the design process. Students learn how to evaluate the best point of leverage within a given clinical need, how to evaluate solutions, and how to balance clinical benefits alongside a viable commercial model. Efforts like Coulter College and training that combines clinical immersion and biodesign will benefit from expansion and a constant focus on underserved disparity populations.

Partnerships between engineering and medicine at programmatic levels constitute another mechanism to provide training and work for engineers in the health disparities arena. Translational medicine, biodesign, and health disparities educators and researchers should collaborate to raise awareness among engineers about racial and ethnic health disparities and to make clinicians and public health experts aware of engineering approaches. Not only will this cross-disciplinary effort enhance the development of clinically relevant solutions, it can serve as a draw for students from diverse backgrounds who are more likely to engage in health disparities explorations [9]. Relating academic studies to skills development for the elimination of disparities in the communities that students come from often is a strong motivator for students [10].

The engineering response to COVID-19 has been strong, as outlined above, yet the disproportionate impact of the pandemic on communities of color calls for engineering solutions that can also address health disparities.

## Call to Action

Engineers are poised and ready to address COVID-19. Deploying that talent with an eye toward elimination of disparities can and will have lasting implications related to the COVID-19 contagion and its disproportionate toll on Blacks. Engineers must work hand-in-hand with physicians to expand access to testing, protective equipment, and therapies. At present, our engineering workforce lacks sufficient diversity. Efforts to recruit and retain a diverse cadre must be taken on with a renewed sense of urgency.
